# Hypoxia regulates IL-17A secretion from nasal polyp epithelial cells

**DOI:** 10.18632/oncotarget.22189

**Published:** 2017-10-31

**Authors:** Qian Xiu, Chenfei Kong, Yiyao Gao, Yang Gao, Jichao Sha, Na Cui, Dongdong Zhu

**Affiliations:** ^1^ Department of Otolaryngology-Head and Neck Surgery, China-Japan Union Hospital, Jilin University, Changchun, China; ^2^ Department of Scientific Research Center, China-Japan Union Hospital, Jilin University, Changchun, China; ^3^ Department of Clinical Medicine, Bethune Medical College, Jilin University, Changchun, China

**Keywords:** chronic rhinosinusitis with nasal polyps, hypoxia, epithelial cells, hypoxia inducible factors, interleukin 17A

## Abstract

Hypoxia creates a microenvironment conducive to polypogenesis by regulating immune responses of the nasal polyp (NP) epithelium. We explored the immunocompetence of NP and control epithelial cells in response to hypoxia, to investigate potential relationships with polypogenesis. Three groups of tissue samples were collected: inferior turbinate (IT)and NP from individuals with chronic rhinosinusitis with NPs (CRSwNP), and control IT. A positive relationship was detected between HIF1α, HIF2α protein expression in epithelial cells and endoscope score in NP samples, while there was a negative correlation between HIF1α expression and degree of eosinophil infiltration. Epithelial IL-17A expression was lower in NPs than in IT samples from either controls or patients with CRSwNP. Primary human nasal epithelial cells were cultured under hypoxic or normoxic conditions. Enzyme-linked immunosorbent assays demonstrated decreased IL-17A expression upon prolonged exposure to hypoxia in both IT and NP samples from patients with CRSwNP, while IL-17A increased in control IT epithelial cells; correlation and time-dependency were observed between HIF1α and IL-17A expression in both IT and NP samples from patients with CRSwNP. These observations suggest that hypoxia is involved in the pathogenesis of NPs through regulation of IL-17A secretion and HIF1α and HIF2α expression in the NP epithelium.

## INTRODUCTION

Chronic rhinosinusitis with nasal polyps (CRSwNP) is a common otolaryngological disease, and the recommended therapeutic regimen usually involves surgery and glucocorticoid treatment; however, high recurrence rates (approximately 53%–60%)remain an unsolved problem [1–3]. Although several hypotheses have been advanced to describe the pathogenesis of CRSwNP, including allergic reaction, the microenvironment of the middle meatus, aspirin intolerance, heredity, dysfunction of the nasal mucosa cilia, and bacterial super antigens [4], no mechanism has been confirmed, and the pathogenesis of CRSwNP remains unclear. Nevertheless, the hypothesis suggesting that the middle meatus has ability to generate a hypoxic microenvironment, owing to its specialized anatomical structure, has been widely accepted. Studies have focused on localized immune responses, since the nasal epithelium is the first line of defense against the external environment, and the immune response to external stimuli involves the synthesis and secretion of cytokines [5], which cause downstream inflammatory responses leading to disease. Various biological therapies have been implemented to manage CRSwNP, particularly in combination with asthma [6]. The identification of suitable cytokines to act as biomarkers may permit the development of a practical treatment options for CRSwNP and elucidate the mechanism underlying polypogenesis.

Hypoxia inducible factors (HIFs) are proteins involved in the regulation of oxygen homeostasis. In hypoxic environments, HIF1α and HIF2α, can bind to hypoxia responsive elements in regulatory regions of target genes to control their transcription levels [7]. Increased expression of HIF1α and its downstream mediators regulate VEGF protein levels in nasal polyps (NPs)compared with normal turbinate tissue [8, 9];however, the roles of HIF1α and HIF2α in the pathogenesis of CRSwNP have yet to be elucidated. Although recent studies have focused on NP tissue and HIF1α[8] [9] [10], few studies have investigated nasal epithelial cell reactions during hypoxia. Thus, we investigated epithelial cells, which are the first barrier to sense changes in oxygen content, to ascertain their role under hypoxic conditions and identify important factors in NP pathogenesis.

Eosinophilic and non-eosinophilic CRSwNP are associated with T helper (Th) 2 and Th1/Th17 cytokines, respectively [11]. Previous studies have revealed that levels of Th1 and Th2 associated cytokines, including interleukin(IL)-5, IFN-γ, and thymic stromal lymphopoietin (TSLP), correlate with those of HIF1α. Specifically, a positive correlation was identified between IL-5 and HIF1α in a murine model of allergic rhinitis [12, 13], and the loss of HIF2α in the alveolar epithelium of mouse lung leads to enhanced eosinophilic inflammation [14]. Direct involvement of HIF1αwas observed in UVB-mediated TSLP induction in human keratinocytes [15], and an IFN-γ-induced increase in HIF1α is associated with activation of NF-κB [16]. However, alterations in Th cytokines in CRSwNP epithelial cells in response to hypoxia, and particularly the cellular and molecular mechanisms involved, remain poorly understood.

IL-17A plays important roles in immunological reactions and inflammatory processes. It promotes circulating polymorphonuclear neutrophil numbers by blocking neutrophil apoptosis [17]. IL-17A also recruits neutrophils by inducing the production of nitric oxide and endothelial chemokines, leading to an increase in vascular endothelial permeability [18, 19]. IL-17A expression also differs significantly between NPs and normal nasal mucosa [20]. In a long-term animal model of CRSwNP [18], nasal lavage detected decreased IL-17A levels. Moreover, IL-17 promotes proliferation of lymphatic endothelial cells, growth of lymphatic vessels, and decreased lymphatic return, leading to edema in tissues, which may explain the edema observed in NP tissue [21].

We performed immunohistochemistry on NP and inferior turbinate (IT) specimens to detect IL-5, TSLP, IL-17A, interferon (IFN)-γ, IL-17AR, HIF1α, and HIF2α. We also mimicked a hypoxic microenvironment in cultured epithelial cells and measured IL-5, TSLP, IL-17A, and IFN-γ levels using enzyme-linked immunosorbent assays (ELISAs). Additionally, HIF1α and HIF2α expression levels were investigated by western blotting, to explore the roles of these factors in NP development and elucidate the relationship between them. We also wanted to explore a new potential mechanism how hypoxia leads to CRSwNP and may find a new target for CRSwNP treatment.

## RESULTS

### HIF1α and HIF2α expression in epithelial cells of NP tissue

To investigate HIF1α and HIF2α expression in NPs and control nasal mucosa, we collected IT biopsies from both controls and patients with CRSwNP, and NP samples from patients with CRSwNP for immunohistochemistry, generating three groups of samples: control inferior turbinate (IT (CONTROL)), IT from patients with CRSwNP (IT (CRS)), and nasal polyps from patients with (NP (CRS)). Moreover, the NP (CRS) group was further separated into eosinophilic and non-eosinophilic NP (CRS) groups.

**Figure 1:**
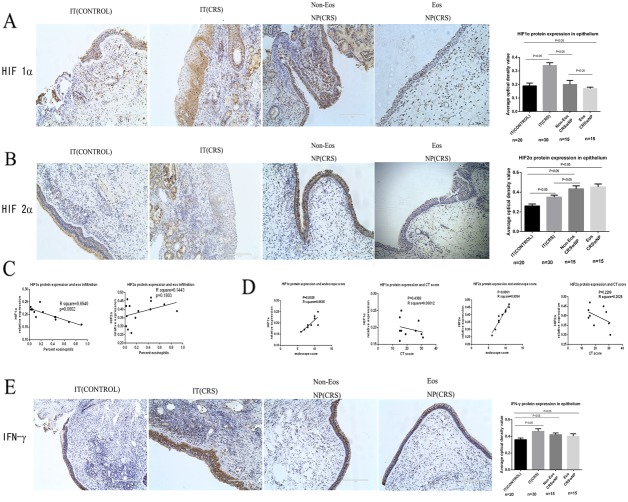
A, B, E show protein expression in epithelial cells quantified by immunohistochemistry (original magnification: 200×) and representative photomicrographs depicting protein immunostaining **(A)** HIF1α protein expression in epithelial cells and the lamina propria in the inferior turbinate (IT) of control, inferior turbinate (IT) of chronic rhinosinusitis with nasal polyps (CRS), eosinophilic (Eos) and noneosinophilic (Non-Eos) chronic rhinosinusitis with nasal polyps NP(CRS) groups. **(B)** HIF2α protein expression in epithelial cells and the lamina propria in the four groups. **(C)** Correlation analysis between HIF1α, HIF2α and eosinophil infiltration degree. **(D)** Correlation analysis between HIF1α, HIF2α and endoscope, CT score in NP(CRS) groups. **(E)** IFN-γ protein expression in epithelial cells and the lamina propria in four groups.

HIF1α and HIF2α were both expressed in nasal epithelial cells, endothelial cells, and glandular epithelial cells, as well as some inflammatory cells (Figure 1A). HIF1α staining in epithelial cells was more intense in the IT (CRS) group, with an integrated optical density(IOD) score of 0.34±0.02. HIF1α expression was lower in both the IT (CONTROL)and NP (CRS) groups compared with the IT (CRS) group. The IOD score of the eosinophilic NP (CRS) group was 0.17±0.01; in contrast, that of the non-eosinophilic NP (CRS) group was 0.20±0.03 which was significantly different. The IOD score of the IT (CONTROL) group was 0.19±0.02, which was significantly higher than levels in the eosinophilic, but not the non-eosinophilic NP (CRS) groups. HIF2α staining in epithelial cells was more intense in the NP group, compared with the IT(CONTROL) and IT(CRS) groups (Figure 1B). The average IOD score of the eosinophilic NP(CRS) group was 0.45±0.03 and that of the non-eosinophilic NP (CRS) group was 0.43±0.03, which was not significantly different. However, both the IT (CONTROL) and IT (CRS) groups exhibited lower values of 0.26±0.02 and 0.35±0.02, respectively, which were significantly different from one another.

We found that HIF1α protein expression levels differed between the eosinophilic and non-eosinophilic NP (CRS) groups. Thus, we performed analysis of the relationship between the degree of eosinophil infiltration and HIF1α protein expression and observed a significant negative correlation between these two phenomena(Figure 1C);however, no significant correlation was observed between HIF2α levels and degree of eosinophil infiltration. Both HIF1α and HIF2α protein expression levels were significantly positively correlated with endoscope score in the NP (CRS) group; however, no significant relationships were identified between CT score and expression of these two proteins in the NP(CRS) group(Figure 1D).

### IL-5, TSLP, and IFN-γ expression differed among the three groups

To evaluate the expression of different cytokines in NP, IT (CONTROL), and IT (CRS) epithelial cells, we detected TH1-, TH2-, and TH17-associated cytokines by immunohistochemistry. Staining for IFN-γ(a TH1 cytokine) indicated that the most intense expression occurred in epithelial cells from the IT (CRS) group (0.46±0.03), while its expression was lower in the non-eosinophilic NP (CRS) group (0.42±0.02) (Figure 1E) than in the IT (CRS), but more intense than that in the IT (CONTROL) group (0.36±0.02). The difference between the non-eosinophilic NP (CRS) and IT (CRS) groups was not significant; however, significant differences were noted compared to the IT (CONTROL) group. IFN-γ protein levels were higher in non-eosinophilic NP (CRS)than eosinophilic NP (CRS) epithelial cells(0.40±0.03), although this difference was not significant. In the NP matrix, IFN-γ-positive cells were observed in NP and IT (CRS) group tissues, and more intense staining was noted in the latter group compared to the former, particularly in the glandular epithelial cells.

**Figure 2:**
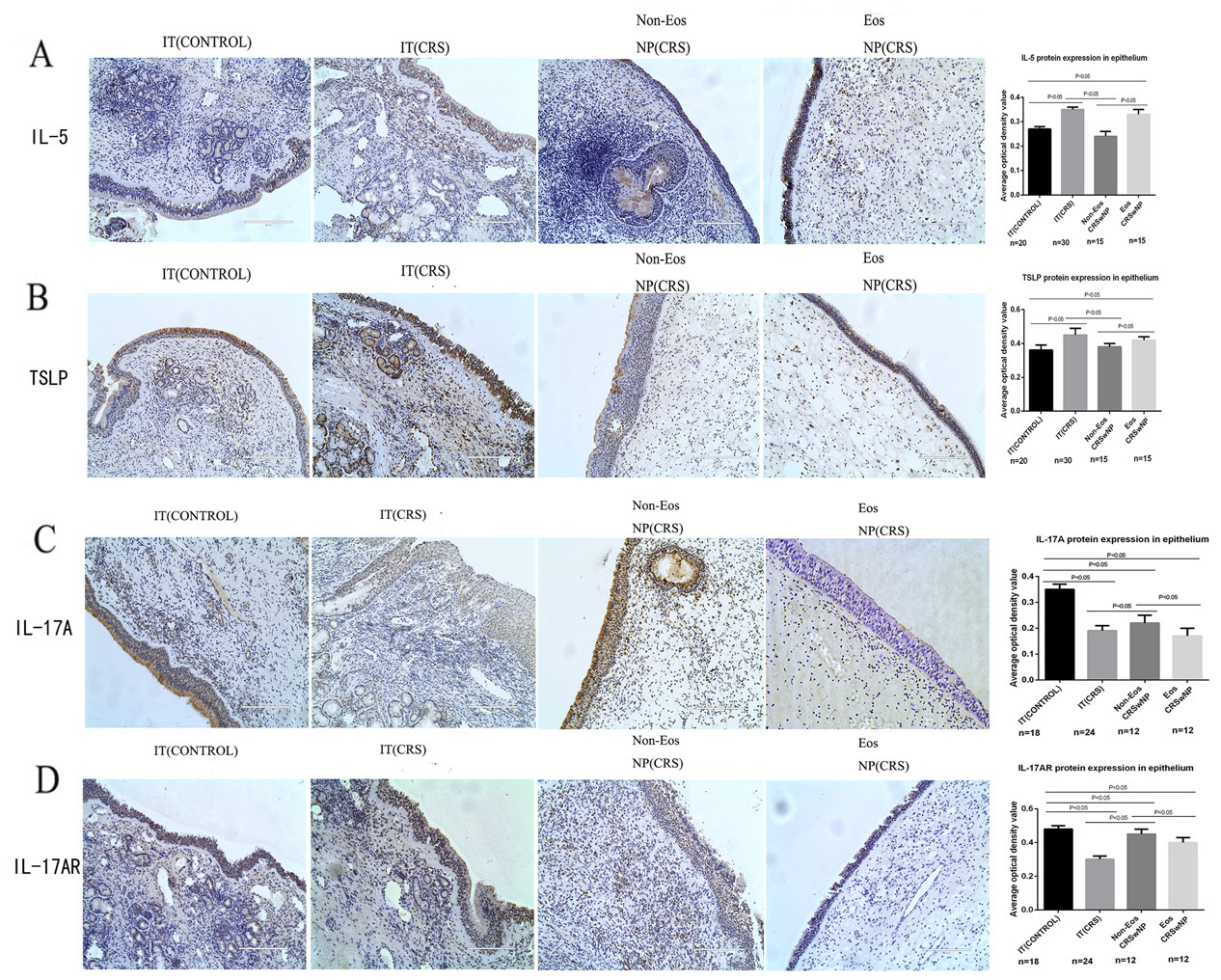
IL-5, TSLP, IL-17A and IL-17A receptor (IL-17AR)expression in epithelial cells derived from all four groups: inferior turbinate (IT) of control, inferior turbinate (IT) of chronic rhinosinusitis with nasal polyps (CRS)eosinophilic (Eos) and noneosinophilic (Non-Eos) chronic rhinosinusitis with nasal polyps NP(CRS) groups A-C show protein expression in epithelial cells quantified by immunohistochemistry (original magnification: 200×) and representative photomicrographs depicting protein immunostaining. **(A)** IL-5 protein expression in epithelial cells and the lamina propria. **(B)** TSLP protein expression in epithelial cells and the lamina propria. **(C)** IL-17A protein expression in epithelial cells and the lamina propria. **(D)** IL-17AR protein expression in epithelial cells and the lamina propria.

We also detected the expression of IL-5(a TH2 cytokine) in the three sample groups and found differences between them regarding the location and quantity of IL-5-positive cells. IL-5 staining in epithelial cells was most intense in the IT (CRS) group (0.35±0.01) (Figure 2A), while in the non-eosinophilic NP (CRS) (0.24±0.02) and IT (CONTROL) (0.27±0.01) groups, there were fewer positive epithelial cells than in the IT (CRS) group. Moreover, IL-5 expression was significantly more intense in the eosinophilic than the non-eosinophilic NP (CRS) group. There were no significant differences in IL-5 levels between the non-eosinophilic NP (CRS) and IT (CONTROL) groups; however, significant differences were observed between the IT (CRS) group and these two groups. In the NP matrix, IL-5-positive cells were noted in NP and IT (CRS) group tissues, and more intense staining was observed in the latter than in the former.

TSLP staining was also most prominent in epithelial cells of the IT (CRS) group (0.45±0.04), and was less intense in the eosinophilic NP (CRS) group (0.42±0.02) (Figure 2B). The smallest number of TSLP-positive epithelial cells were observed in the IT (CONTROL)(0.36±0.03) and non-eosinophilic NP (CRS)(0.38±0.02)groups, and there was no significant difference between these two groups. There were also no significant differences between the eosinophilic NP (CRS)and IT (CRS) groups. TSLP protein levels were more intense in the eosinophilic than the non-eosinophilic NP (CRS) groups.

Overall, our results demonstrate that non-eosinophilic NP epithelial cells expressed TSLP and IL-5 at the same levels as those observed in IT (CONTROL) epithelial cells, while IFN-γ expression differed only slightly among the groups. In contrast, IFN-γ, TSLP, and IL-5 staining levels were more intense in eosinophilic NP than in IT (CONTROL) epithelial cells.

### IL-17A and IL-17AR expression levels were significantly elevated in IT (CONTROL)samples compared with those from other groups

We next sought to characterize IL-17A expression in epithelial cells from all three groups by immunohistochemical analysis. IL-17A-positive cells were observed in the stroma, glandular epithelium, vessel endothelium, and nasal mucosa epithelium in biopsies from the three groups (Figure 2C). IL-17A expression in nasal epithelial cells was most intense in the IT (CONTROL) group (0.35±0.02) relative to the IT (CRS)(0.19±0.02) and the eosinophilic NP (CRS)(0.17±0.03) groups, and the difference was significant(p<0.05). IL-17A protein levels was (0.22±0.03) in the non-eosinophilic NP (CRS)group which was significantly different from those of the other groups. Based on these results, we infer that epithelial cells from the IT (CONTROL) group secrete more IL-17A than those from the NP group.

To further evaluate IL-17A expression in epithelial cells and investigate its possible function in the nasal mucosa matrix, we performed immunohistochemistry to detect IL-17AR, the receptor for IL-17A. Expression of IL-17AR is thought to positively correlate with that of IL-17A [22]. Samples in the IT (CONTROL) group exhibited the most IL-17AR-positive epithelial cells (0.48±0.02), followed by the non-eosinophilic NP (CRS) group (0.45±0.03), and finally the IT (CRS) group (0.30±0.03), and expression differed significantly among the three groups. IL-17AR protein expression was slightly, but significantly, lower in the eosinophilic NP (CRS) group (0.40±0.03) than in both the IT (CONTROL) and non-eosinophilic NP (CRS) groups. IL-17AR staining was observed in epithelial cells, macrophages, endothelial glandular epithelial cells, fibroblasts, and other inflammatory cells. In the NP and IT (CONTROL) groups (Figure 2D), positive cells were concentrated in the epithelial cells, vessel endothelial cells, fibroblasts, and macrophages; however, in the IT (CRS) group, positive cells were found not only among epithelial cells, vessel endothelial cells, and macrophages, but also in glandular epithelial cells. Thus, IL-17A may have an important role in inflammation of the nasal mucosa.

### HIF1α is strongly expressed in NP epithelial cells in response to hypoxic conditions

**Figure 3:**
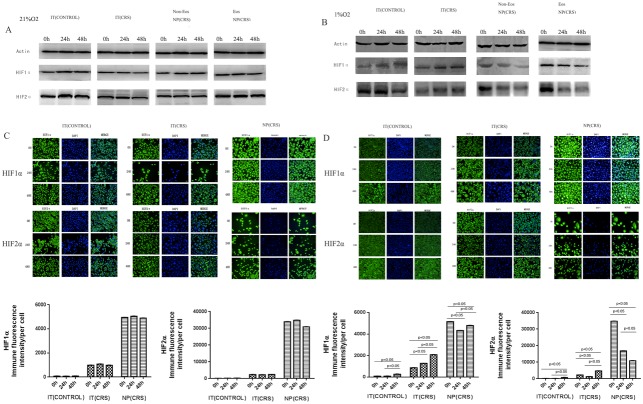
After stimulated with normoxia(21% O_2_) and hypoxia (1% O_2_), proteins from epithelial cells of all four groups were immunoblotted with specific antibodies: HIF1α and HIF2α **(A)** HIF1α and HIF2α protein expression in epithelial cells exposure to normoxia(21% O_2_) condition. **(B)** HIF1α and HIF2α protein expression in epithelial cells exposure to hypoxia(1% O_2_) condition. **(C)** After stimulated with normoxia (21% O_2_), HIF1α and HIF2α protein expression in epithelial cells were quantified by performing immunofluorescent staining and high-content analysis (scale bars are 50 um) in the IT(CONTROL), IT(CRS), NP(CRS) groups. Statistic result of immunofluorescent staining and high-content analysis. **(D)** After stimulated with hypoxia (1% O_2_), HIF1α and HIF2α protein expression in epithelial cells were quantified by performing immunofluorescent staining and high-content analysis (scale bars are 50 um) in the three groups. Statistic result of immunofluorescent staining and high-content analysis. IT(CONTROL) = inferior turbinate of control, IT(CRS) = inferior turbinate of CRSwNP, NP(CRS) = nasal polyps.

HIF1α expression is associated with stimulation by hypoxia and our immunochemistry results illustrated that HIF1α is strongly expressed in NP epithelial cells. Therefore, we inferred that NP epithelial cells had developed in an hypoxic environment. The four groups of epithelial cells were cultured under hypoxic and normoxic conditions. By western blotting(Figure 3A), we found that the expression levels of both HIF1α and HIF2α were highly elevated in the NP(CRS) group relative to the two IT groups (control and CRS). Moreover, there were no differences between the eosinophilic and non-eosinophilic NP (CRS) groups with respect to the levels of these two proteins, regardless of whether cells were cultured under normoxic or hypoxic conditions. No significant changes were observed in the expression of HIF1α and HIF2α over time under normoxic conditions; however, culture under hypoxic conditions for 24 and 48 h, led to relatively higher levels of HIF1α and HIF2α in the NP epithelial cells compared with those in the IT (CONTROL) and IT (CRS) groups in a time-dependent manner. While in NP epithelial cells, levels of these proteins decreased at 24 and 48h compared to 0h(Figure 3B). These results were confirmed by immunofluorescent staining and high-content analysis(Figure 3C and 3D). Based on these findings, we conclude that our experiments successfully mimicked the hypoxic microenvironment which facilitates polyp pathogenesis. Importantly, the NP group appeared to be more sensitive to hypoxia compared with the other two groups.

### IL-5, TSLP, and IFN-γ expression did not alter in response to hypoxia

**Figure 4:**
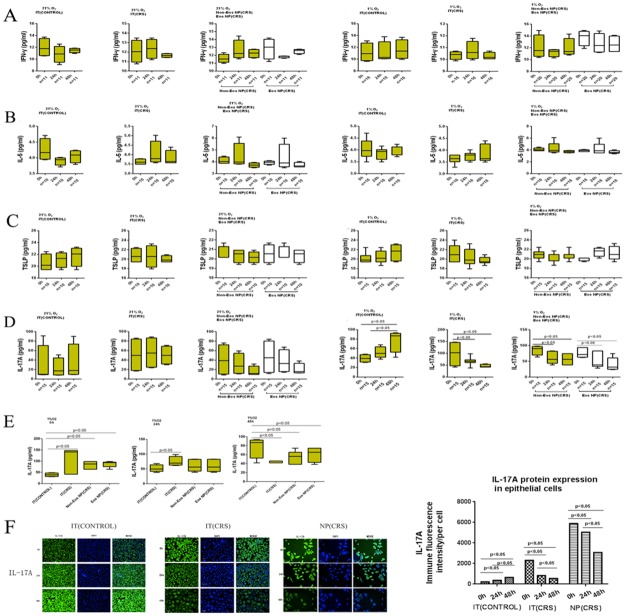
After stimulated with normoxia(21% O_2_) and hypoxia (1% O_2_), protein levels for the various cytokines/chemokines: IFN-γ, IL-5, TSLP and IL-17A were determined by ELISA A-D Left column Epithelial cells were under normoxia exposure. Right column Epithelial cells were under hypoxia exposure. **(A)** IFN-γ secreted from epithelial cells after stimulation for 0 hours, 24 hours, and 48 hours. **(B)** IL-5 secreted from epithelial cells after stimulation for 0 hours, 24 hours, and 48 hours. **(C)** TSLP secreted from epithelial cells after stimulation for 0 hours, 24 hours, and 48 hours. **(D)** IL-17A secreted from epithelial cells after stimulation for 0 hours, 24 hours, and 48 hours. **(E)** After stimulated with hypoxia (1% O_2_), different IL-17A secretion levels from four types of epithelial cells derived from inferior turbinate (IT) of control, inferior turbinate (IT) of chronic rhinosinusitis with nasal polyps (CRS), eosinophilic (Eos) and noneosinophilic (Non-Eos) chronic rhinosinusitis with nasal polyps NP(CRS) groups were examined by ELISA. **(F)** IL-17A protein expression in IT(CONTROL), IT(CRS) and NP(CRS) epithelial cells was quantified by immunofluorescent staining and high-content analysis. (scale bars are 50 μm) Statistic result of immunofluorescent staining and high-content analysis.

To further investigate the responses of the three groups to hypoxia and normoxia, we measured the secretion of IFN-γ, IL-5, and TSLP from cultured epithelial cells under both hypoxic and normoxic conditions by ELISA. IFN-γ levels did not differ between normoxic and hypoxic conditions for the IT (CONTROL), eosinophilic and non-eosinophilic NP (CRS), and IT (CRS) groups(Figure 4A), nor did IL-5 levels differ over time among the three groups under normoxic or hypoxic conditions (24 h) (Figure 4B). Under normoxic conditions, the IT (CRS) group expressed the lowest levels of IL-5 among the three groups (p<0.05) (3.60±0.18 pg/mL). IFN-γ and IL-5 mRNA expression in the epithelial cells from the above groups were consistent with the ELISA results (Supplementary Figure 1A, 1B).

The results of ELISAs indicated no significant differences in TSLP levels between cells from the eosinophilic and non-eosinophilic NP (CRS), IT (CONTROL), and IT (CRS) groups grown under normoxic and hypoxic conditions (Figure 4C).

Thus, during hypoxia, epithelial cells from the NP and IT (CONTROL) groups did not exhibit significant differences in IFN-γ, IL-5, or TSLP secretion, whereas cytokine secretion differed significantly in the IT (CRS) group.

### Epithelial cells from all three groups differed in their levels of IL-17A expression under hypoxia compared with normoxia

We performed ELISAs using supernatants from isolated epithelial cells cultured under hypoxic and normoxic conditions to ascertain the different responses of the three groups to different oxygen conditions. There were no significant differences in IL-17A levels between the eosinophilic and non-eosinophilic NP (CRS), IT (CONTROL), and IT (CRS) groups under normoxia over time (Figure 4E). However, under hypoxic conditions (1% O_2_ for 24 h), the IT (CRS) group produced more IL-17A (74.7.1±14.8 pg/mL) compared with the IT (CONTROL)(52.1±12.3 pg/mL), non-eosinophilic NP (CRS) (60.6±19.7 pg/mL), and the eosinophilic NP (CRS)(60.6±20.9pg/mL)groups (p<0.05). After 48 h exposure to hypoxic conditions, the IT (CONTROL) group produced more IL-17A (77.3±21.7pg/mL) compared with the non-eosinophilic NP (CRS) (52.7±12.0 pg/mL), eosinophilic NP (CRS)(60.9±17.3 pg/mL), and the IT (CRS)(43.1±2.2 pg/mL) groups (p<0.05). There were no significant differences in IL-17A secretion between eosinophilic and non-eosinophilic NP (CRS)groups exposed to hypoxia for the same duration.

We next investigated whether the duration of hypoxia exposure influenced the levels of secretion of IL-17A among the different groups(Figure 4D). IL-17A expression decreased with increased hypoxia exposure time in the IT (CRS) group (p<0.05). IL-17A expression also decreased under hypoxic compared with normoxic conditions in both the eosinophilic and non-eosinophilic NP (CRS) groups (p < 0.05);however, IL-17A levels did not differ significantly between 24 and 48 h of hypoxia exposure. In contrast, IL-17A levels increased significantly during prolonged hypoxia exposure in the IT (CONTROL) group (p<0.05). IL-17A expression in epithelial cells from all three groups was confirmed by immunofluorescent staining and high-content analysis(Figure 4F). After 48 h of hypoxia stimulation, increased IL-17A expression was observed in IT (CONTROL) epithelial cells compared with cells stimulated for 24 h and those grown under normoxic conditions. IL-17A expression decreased with prolonged hypoxia exposure in IT (CRS) epithelial cells. There were no significant differences in IL-17A expression between NP epithelial cells exposed to hypoxia for 48 and 24 h; however, decreased IL-17A expression was noted in comparison to cells cultured under normoxia. The IL-17A mRNA expression levels in epithelial cells from the above groups were with the ELISA results (Supplementary Figure 1C).

### Correlation between HIF1α and IL-17A levels in different oxygen concentrations and clinical characteristic

**Figure 5:**
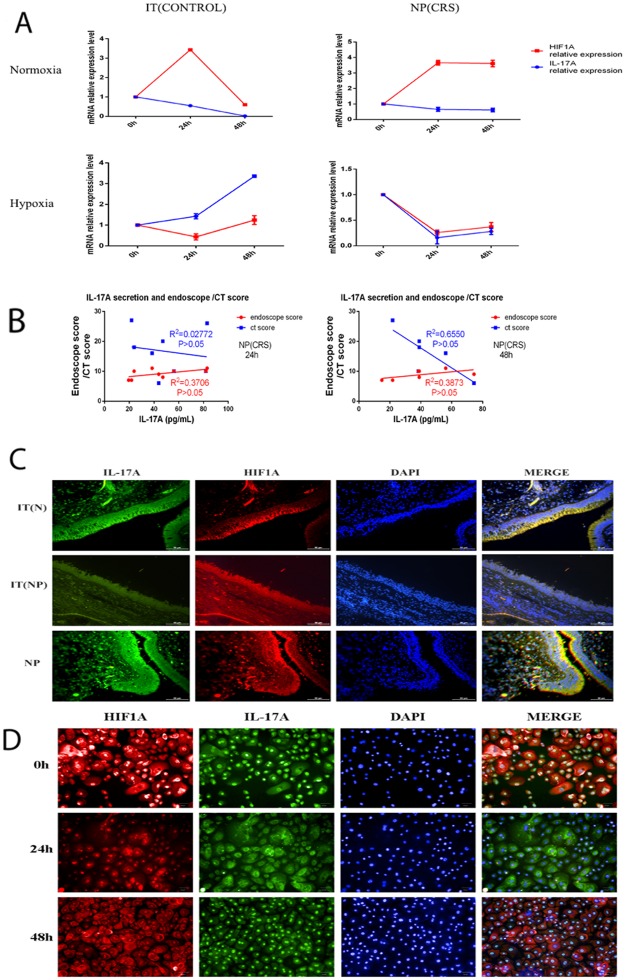
**(A)** In normoxia condition IL-17A and HIF1α mRNA levels were not related as time extended both in control and nasal polyps epithelial cells. In hypoxia condition the two mRNA levels changing trend over time were consistent in the two kinds of epithelial cells. **(B)** Correlation analysis between IL-17A secretion after hypoxia exposure for 24h or 48h in nasal polyps epithelial cells and the same patients’ endoscopic or CT score. **(C)** IL-17A and HIF1α dual labelled immunofluorescence on IT(CONTROL), IT(CRS) and NP(CRS) epithelial cells. The two protein were co-localized. (scale bars are 50 um) **(D)** IL-17A and HIF1α dual labelled immunofluorescence on NP(CRS) epithelial cells after hypoxia exposure for 0h,24h or 48h. (scale bars are 50 um)

Our results illustrated that IL-17A secretion and HIF1α protein levels in nasal epithelial cells decreased under hypoxic conditions. Thus, we compared both IL-17A and HIF1α mRNA levels in NP epithelial cells under hypoxic and normoxic conditions. In NP epithelial cells under normoxic conditions, we found that IL-17A and HIF1α mRNA levels were not correlated over time; however, under hypoxic conditions, changes in levels of the two mRNAs correlated with one another over time. Similar results were observed in normal nasal epithelial cells, under both normoxic and hypoxic conditions (Figure 5A).

Next we wanted to determine the relationship between IL-17A secretion and endoscope and CT scores; however, no significant correlation was found between IL-17A secretion after hypoxia exposure for 24 or 48 h in NP epithelial cells and patient endoscopic or CT scores(Figure 5B). Subsequently, we performed dual labelled immunofluorescence on tissue samples of three groups to investigate co-localization of the expression of HIF1α and IL-17A. Our results indicated that the two proteins co-localized in IT(CONTROL), IT(CRS), and NP (CRS) epithelial cells, and that expression of these two proteins was correlated with one another in these three groups(Figure 5C). We also performed dual labelled immunofluorescence on epithelial cells from NPs, and observed decreased expression of IL-17A and HIF1α in cells exposed to hypoxia compared with those cultured under normoxia (Figure 5D).

## DISCUSSION

Previous studies have noted increased expression of HIF1α and HIF2α in NP tissue compared with healthy nasal mucosa; however, these studies did not specifically investigate epithelial cells. The nasal epithelium is the first line of defense in the nasal microenvironment and initiates immune responses to external stimuli by synthesizing and secreting cytokines [5]. In our study, we observed increased expression of HIF1α and HIF2α in NP epithelial cells, and increased expression of HIF1α was also noted in vessel endothelial cells in NPs. Although Shin and colleagues [23] cultured human nasal epithelial cells under hypoxic conditions, those were cell lines derived from healthy individuals and, therefore, not equivalent to the NP epithelial cells investigated here.

In addition, the previous study did not investigate the effects of exposure to hypoxia for different lengths of time. In our study, all the cells analyzed were primary epithelial cells isolated from controls and patients with CRSwNP cultured under hypoxic or normoxic conditions. Most notably, elevated HIF1α and HIF2α expression was observed in eosinophilic and non-eosinophilic NP epithelial cells compared with the two IT groups. Furthermore, this expression was prominent in NP epithelial cells following exposure to hypoxia for both short (24 h) and longer(48 h) durations. Shin et al. obtained results similar to ours, demonstrating that after 48 h of hypoxia stimulation, HIF1α and HIF2α expression increased in human nasal epithelial cell lines [23];however, they did not include short duration(24 h) exposure to hypoxia. Hence, our study demonstrates the tissue- and time-dependency of HIF1α and HIF2α expression after exposure to hypoxia, where HIF1α and HIF2αlevels decreased in NP epithelial cells. Moreover, expression levels of HIF1α and HIF2α in NP epithelial cells were much higher than those in control nasal epithelial cells, regardless of hypoxia or normoxia. This specificity suggests HIF1α and HIF2α as potential targets in NP pathogenesis.

In Western populations, CRSwNP is often considered to be caused by type 2 inflammation, characterized by elevated levels of type 2 cytokines, such as IL-5 and IL-13, along with eosinophil infiltration. In contrast, in Asian countries, CRSwNP may be considered to be a result of mixed type 1 or type 3 inflammation, exemplified by IL-17 and IFN-γ secretion, which is more neutrophilic than the inflammation observed in NPs from patients from Europe or the United States [24]. However, we consider that previous studies investigating different cytokines secreted by epithelial cells under hypoxia have been insufficient. In this study, hypoxia did not influence the secretion of TSLP, IL-5, or IFN-γ in NP and IT epithelial cells. Although the immunohistochemistry results for IFN-γ suggested differential secretion, the NP group secreted less IFN-γ than the IT (CRS) group, but more than the IT (CONTROL) group. Thus, IFN-γ appears to participate in polypogenesis, but not in response to hypoxia stimulation.

Studies investigating NP tissue have observed higher IL-17A expression in NPs than in healthy nasal mucosa, whereas no significant differences in IL-17A expression were observed between NP and normal nasal epithelial cells [10]. However, the changes demonstrated by epithelial cells upon exposure to hypoxia have not previously been investigated. In our experiments, IL-17A secretion from control epithelial cells increased with prolonged hypoxia exposure, while IL-17A secretion declined in both NP and IT epithelial cells from CRSwNP patients. IL-17A immunohistochemistry, immunofluorescent staining, and high-content analysis also confirmed our hypothesis. Based on these results, IL-17A was the most prominent immune cytokine in NP epithelial cells under hypoxic conditions.

IL-17AR belongs to the IL-17R family, which is primarily expressed by epithelial cells, endothelial cells, and fibroblasts; however, macrophages and dendritic cells are also responsive to IL-17A [25]. In a study by Li and colleagues [22], IL-17A significantly increased IL-17AR expression in cultured polyp epithelial cells in a dose-dependent manner. Similar results were observed in our study; we noted the decreased expression of IL-17AR and IL-17A in both eosinophilic and non-eosinophilic NP epithelial cells compared with controls, and a positive correlation between IL-17AR and IL-17A expression was identified. The significant differences in IL-17A expression observed between the IT (CONTROL)and NP groups may reflect differential inflammatory responses related to polypogenesis. IL-17A is best known for its participation in neutrophil recruitment and survival [26]. Hypoxia stimulation of NP epithelial cells may lead to a decline in IL-17A secretion, which could influence their ability to recruit neutrophils and block apoptosis. The consequent decrease in numbers of neutrophils could lead to suppression of the TH1 response. Additional experiments are needed to test this hypothesis. Thus, hypoxia may be involved in polyp pathogenesis through regulation of IL-17A secretion.

In our experiments, exposure of NP epithelial cells to prolonged hypoxia resulted in correlated changes in HIF1α expression and IL-17A secretion. IL-17A and HIF1α can interact with one another, particularly under hypoxic conditions [27, 28]. We found that both HIF1α and IL-17A expression increased in control epithelial cells exposed to prolonged hypoxia exposure, illustrating a positive correlation between HIF1α and IL-17A expression. HIF1α regulates the balance between T regulatory (Treg) and T helper 17(TH17) cell differentiation [29, 30], and deficiency of HIF1α is associated with diminished TH17 and increased Treg cell numbers. HIF1α may inhibit recruitment of TH17 cells by decreasing IL-17A expression in NPs. An imbalance of TH17 and Treg cells has been reported in NPs [31, 32]. Alternatively, decreased IL-17A secretion may be related to edema in NP tissue, sinceIL-17 directly promotes growth of lymphatic vessels and proliferation of lymphatic endothelial cells in cultured primary human corneal epithelial and lymphatic microvascular endothelial cells [21]. Tissue edema is related to decreased lymphangiogenesis because of disturbed fluid circulation. Sub-epithelial edema is found in NPs; however, no lymphatic vessels have been found in this context [33]. Decreased IL-17A expression may result in reduced lymphangiogenesis, and consequent edema in NPs; however, in our research, we observed a significant sharp decrease in HIF2α expression in NP epithelial cells under hypoxia over time. There was a similar decrease in IL-17A secretion; however, there are no reports in the literature of a relationship between IL-17A and HIF2α. Hence, the relationship between HIF1α, IL-17A, HIF2α, and lymphangiogenesis requires further investigation.

Our study illustrates that NP epithelial cells secrete less IL-17A under hypoxic compared with normoxic conditions. More interestingly, IT epithelial cells from patients with CRSwNP showed the same changes in IL-17A over time, where, as the period of exposure to hypoxia increased, secretion of IL-17A clearly decreased. However, IT epithelial cells from controls exhibited the opposite effect, with IL-17A secretion increasing in response to increased exposure to hypoxia. Moreover, although IL-17A secretion in the IT epithelial cells showed the same tendency in IT and NP epithelial cells from patients with CRSwNP, HIF1α expression levels differed between the two groups of CRSwNP samples. Under hypoxic conditions, IT epithelial cells from both CRSwNP and control samples exhibited similar changes in HIF1α expression over time. The observation of differential expression of IL-17A and HIF1α among the three groups prompts us to hypothesize that both genetic and environmental components may influence the risk of CRSwNP. Extensive genealogical database analysis confirmed that there is a familial component to risk of CRSwNP, which may include both genetic and environmental factors [34]. To clarify these phenomena, further research into epigenetic modification and genetic expression profiles in CRSwNP is required.

**Figure 6:**
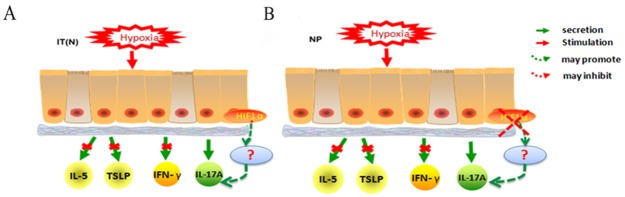
**(A)** Hypoxia stimulated IT(N) epithelial cells, no differences in IL-5, TSLP, IFN-γ secretion when compared with normoxia. IL-17A secretion appeared changes under hypoxia. HIF1α promoted IL-17A secretion in epithelial cells under hypoxia. **(B)** Hypoxia stimulated NP epithelial cells, no differences in IL-5, TSLP, IFN-γsecretion stimulated by hypoxia when compared with normoxia. IL-17A secretion appeared changes under hypoxia. Decreased HIF1α inhibited IL-17A secretion in epithelial cells under hypoxia.

In conclusion, our study demonstrates that NP epithelial cells are more sensitive to hypoxia compared with normal nasal epithelial cells, and that HIF1α and HIF2α expression increases in NP epithelial cells exposed to hypoxia for longer periods of time. Under hypoxic conditions, changes in the immunocompetence of NP epithelial cells primarily manifested as decreased secretion of IL-17A, compared with cells cultured under normoxic conditions. Levels of IL-5, TSLP, and IFN-γ were not affected by hypoxia in nasal epithelial cells. A correlation was observed between HIF1α expression and IL-17A secretion in NP epithelial cells exposed to hypoxia. We suspect that hypoxia induces changes in TH17 immunocompetence, which may play an important role in the early stages of polyp pathogenesis(Figure 6); additional research is required to investigate this hypothesis further.

## MATERIALS AND METHODS

### Study population and experimental groups

Patient characteristics are included in Table 1. The Institutional Review Board of China-Japan Union Hospital of Jilin University approved this study, and all methods were performed in accordance with the relevant guidelines and regulations. All controls and subjects with CRSwNP provided signed informed consent and were recruited from the Department of Otolaryngology Clinic at the China-Japan Union Hospital of Jilin University. All subjects met the criteria for CRS, as defined by the American Academy of Otolaryngology-Head and Neck Surgery Chronic Rhinosinusitis Task Force [35]. Based on their medical histories, none of the patients suffered from aspirin intolerance, allergic rhinitis, or asthma.

**Table 1:** Characteristics of patients and controls

	CRSwNP		Control IT (CONTROL)
	NP	IT (CRS)	
Sex ratio (F:M)	1:1.87	1:1.87	1:1.83
Age (x±s)	47.64±16.00	47.64±16.00	39.8±8.20
Patients with smoking exposure, n (%)	10(31)	10(31)	6(20)
Patients with allergic rhinitis, n (%)	0	0	0
Patients with asthma, n (%)	0	0	0
Bilateral VAS score, median (IQR)	17.55±4.34	17.55±4.34	8.0±3.23
Bilateral CT score, median (IQR)	18.03±9.67	18.03±9.67	0±2.0

CRSwNP, chronic rhinosinusitis with nasal polyps; NP, nasal polyps; IT(CRS): inferior turbinate from patients with CRSwNP; IT(CONTROL), inferior turbinate from controls. F, female; M, male; IQR, interquartile range; VAS, visual analog scale; CT, computed tomography.

We separated biopsies and primary cells into three groups: control inferior turbinate (IT (CONTROL)), IT from patients with CRSwNP (IT (CRS)), and nasal polyps from patients with CRSwNP (NP(CRS)). Moreover, the NP (CRS) group were further separated into eosinophilic and non-eosinophilic NP (CRS) groups. Normal IT epithelial cells were collected from 17 controls, while IT and NP samples were obtained from 32 CRSwNP patients. Sixteen samples were successfully cultured from the IT (CONTROL) group, with 30 cultures achieved from the IT (CRS) and NP (CRS) groups (Table 2).

**Table 2:** Numbers of samples from each group included in different experiments

Experiment type	CRSwNP		ControlIT (CONTROL)
	NP	IT (CRS)	
Primary cells cultured	32	31	17
Immunohistochemistry	30	30	20
ELISA	20	16	16
Western blot	26	25	16
quantitative RT-PCR	6	6	6
high-content/high-throughput analysis	10	10	6

RT-PCR, reverse transcription polymerase chain reaction

### Nasal biopsies and primary nasal epithelial cell culture

Primary NP epithelial cells and IT epithelial cells were freshly isolated from controls and patients with CRSwNP. Biopsies of nasal epithelial cell sheets from the IT of healthy volunteers were collected. Cells were then proliferated for seven to ten days on collagen-coated 3.5cm^2^ dishes (Thermo Scientific, Suzhou, China) in bronchial epithelial cell basal medium (BEGM), and then subjected to fetal bovine serum starvation. Once confluent, cells were trypsinized and re-seeded evenly on 3.5cm^2^dishes (Thermo Scientific, Suzhou, China) coated with human collagen type IV (Sigma, Saint Louis, USA). When cells reached 80% confluence, they were resuspended in BEGM and cultured further for the indicated amount of time (0, 24, or 48 h) in a HERA CELL 150i CO_2_ incubator (Thermo Scientific, USA) with the appropriate oxygen concentrations for hypoxia (1% O_2_) or normoxia (21% O_2_).

### Histological analysis and immunohistochemistry

Paraffin sections were stained with HE and then observed by two independent physicians. The number of eosinophils was counted at high power (HP) magnification (400×), and 10 HP fields were randomly selected and analyzed. Referring to the method of Liu et al. [36], CRSwNP samples were classified as eosinophilic when the percentage of eosinophils exceeded twice the SD of the mean of controls (4.8% + 2×2.12%= 9.04%; therefore, 9% was chosen as the cut-off value).

All monoclonal antibodies (anti-IL-17A, anti-HIF1α, anti-HIF2α, anti-IL-17A receptor, anti-IFN-γ, and anti-TSLP) were purchased from Abcam (Cambridge, MA, USA). An anti-IL-5 polyclonal antibody was purchased from Novus Biologicals. Details of the experimental methods are included in the Supplemental material. Image Pro-Plus 6.0 software was used to analyze images. All images are taken at exactly the same conditions using the same microscope and camera ;image brightness was normalized to background levels. We selected epithelial layer as area of interest (AOI) for measurements. Using Image Pro Plus, the images were changed to grayscale digital images to test total optical density (OD) and calculate the area of AOI; then calculated the mean OD using total OD/area of AOI. The mean OD represents the density of dye staining and reflects the content of the target protein.

### ELISA

Cell culture supernatants were assayed for IL-17A, IL-5, TSLP, and IFN-γ levels using ELISA kits purchased from R&D Systems (Minneapolis, MN USA). Data are expressed as pg/mL.

### Western blotting

Proteins from epithelial cell extracts were denatured and separated by sodium dodecyl sulfate-polyacrylamide gel electrophoresis, electroblotted onto polyvinylidene difluoride membranes (Bio-Rad, Hercules, CA), blocked, and incubated overnight at 4°C with primary anti-HIF1α and anti-HIF2α antibodies. Western blot quantification was performed using an Odyssey infrared imaging system (Gene Company).

### Immunofluorescent staining and imaging

Immunofluorescent staining of assay plates was carried out according to the method of Martin et al [37]. Anti-HIF1α, anti-HIF2α, and anti-IL-17Aprimary antibodies were used(1:50). Details of the experimental method are included in the Supplemental material. Images were analyzed using Columbus 2.2 software (PerkinElmer).

### Quantitative RT-PCR

Quantitative RT-PCR was performed with specific primers purchased from Sangon (Sangon Biotech, Shanghai, China). Relative gene expression was calculated using the 2(-DDCT) method. Additional details are provided in the online supplement.

### Statistics

All data are reported as medians with 25%–75% interquartile ranges or means ±SEM. Differences among groups were analyzed by 2-way ANOVA, Mann-Whitney U-test, or Student's t-test. A p value < 0.05 was considered significant.

## SUPPLEMENTARY MATERIALS FIGURE



## References

[1] Wynn R, Har-El G (2004). Recurrence rates after endoscopic sinus surgery for massive sinus polyposis. Laryngoscope.

[2] Tosun F, Yildizoglu U, Polat B, Durmaz A (2014). Types of endoscopic endonasal resections for sinonasal malignancies. J Craniofac Surg.

[3] Morrissey DK, Bassiouni A, Psaltis AJ, Naidoo Y, Wormald PJ (2016). Outcomes of modified endoscopic Lothrop in aspirin-exacerbated respiratory disease with nasal polyposis. International forum of allergy & rhinology.

[4] Wackym PA, Snow JB (2016). Ballenger's otorhinolaryngology : head and neck surgery.

[5] Comer DM, Elborn JS, Ennis M (2014). Inflammatory and cytotoxic effects of acrolein, nicotine, acetylaldehyde and cigarette smoke extract on human nasal epithelial cells. BMC Pulm Med.

[6] Bachert C, Mannent L, Naclerio RM, Mullol J, Ferguson BJ, Gevaert P, Hellings P, Jiao L, Wang L, Evans RR, Pirozzi G, Graham NM, Swanson B (2016). Effect of Subcutaneous Dupilumab on Nasal Polyp Burden in Patients With Chronic Sinusitis and Nasal Polyposis: A Randomized Clinical Trial. JAMA.

[7] Zagorska A, Dulak J (2004). HIF-1: the knowns and unknowns of hypoxia sensing. Acta Biochim Pol.

[8] Chien CY, Tai CF, Ho KY, Kuo WR, Chai CY, Hsu YC, Wang LF (2008). Expression of hypoxia-inducible factor 1alpha in the nasal polyps by real-time RT-PCR and immunohistochemistry. Otolaryngol Head Neck Surg.

[9] Jiang S, Dong Z, Zhu D, Yang Z (2002). Hypoxia effects on vascular endothelial growth factor derived epithelial cells of nasal polyps. [Article in Chinese]. Zhonghua Er Bi Yan Hou Ke Za Zhi.

[10] Jiang XD, Li GY, Li L, Dong Z, Zhu DD (2011). The characterization of IL-17A expression in patients with chronic rhinosinusitis with nasal polyps. Am J Rhinol Allergy.

[11] Fokkens WJ, Lund VJ, Mullol J, Bachert C, Alobid I, Baroody F, Cohen N, Cervin A, Douglas R, Gevaert P, Georgalas C, Goossens H, Harvey R (2012). European Position Paper on Rhinosinusitis and Nasal Polyps 2012. Rhinol Suppl.

[12] Jang TY, Park CS, Kim KS, Heo MJ, Kim YH (2014). Benzaldehyde suppresses murine allergic asthma and rhinitis. Int Immunopharmacol.

[13] Mo JH, Kim JH, Lim DJ, Kim EH (2014). The role of hypoxia-inducible factor 1alpha in allergic rhinitis. Am J Rhinol Allergy.

[14] Proper SP, Saini Y, Greenwood KK, Bramble LA, Downing NJ, Harkema JR, Lapres JJ (2014). Loss of hypoxia-inducible factor 2 alpha in the lung alveolar epithelium of mice leads to enhanced eosinophilic inflammation in cobalt-induced lung injury. Toxicol Sci.

[15] Jang Y, Jeong SH, Park YH, Bae HC, Lee H, Ryu WI, Park GH, Son SW (2013). UVB induces HIF-1alpha-dependent TSLP expression via the JNK and ERK pathways. J Invest Dermatol.

[16] Yang S, Yu M, Sun L, Xiao W, Yang X, Sun L, Zhang C, Ma Y, Yang H, Liu Y, Lu D, Teitelbaum DH, Yang H (2014). Interferon-gamma-induced intestinal epithelial barrier dysfunction by NF-kappaB/HIF-1alpha pathway. J Interferon Cytokine Res.

[17] Schwarzenberger P, Huang W, Ye P, Oliver P, Manuel M, Zhang Z, Bagby G, Nelson S, Kolls JK (2000). Requirement of endogenous stem cell factor and granulocyte-colony-stimulating factor for IL-17-mediated granulopoiesis. J Immunol.

[18] Konig K, Klemens C, Haack M, Nicolo MS, Becker S, Kramer MF, Groger M (2016). Cytokine patterns in nasal secretion of non-atopic patients distinguish between chronic rhinosinusitis with or without nasal polys. Allergy Asthma Clin Immunol.

[19] Kolls JK, Linden A (2004). Interleukin-17 family members and inflammation. Immunity.

[20] Saitoh T, Kusunoki T, Yao T, Kawano K, Kojima Y, Miyahara K, Onoda J, Yokoi H, Ikeda K (2010). Role of interleukin-17A in the eosinophil accumulation and mucosal remodeling in chronic rhinosinusitis with nasal polyps associated with asthma. Int Arch Allergy Immunol.

[21] Chauhan SK, Jin Y, Goyal S, Lee HS, Fuchsluger TA, Lee HK, Dana R (2011). A novel pro-lymphangiogenic function for Th17/IL-17. Blood.

[22] Xia W, Bai J, Wu X, Wei Y, Feng S, Li L, Zhang J, Xiong G, Fan Y, Shi J, Li H (2014). Interleukin-17A promotes MUC5AC expression and goblet cell hyperplasia in nasal polyps via the Act1-mediated pathway. PLoS One.

[23] Shin HW, Cho K, Kim DW, Han DH, Khalmuratova R, Kim SW, Jeon SY, Min YG, Lee CH, Rhee CS, Park JW (2012). Hypoxia-inducible factor 1 mediates nasal polypogenesis by inducing epithelial-to-mesenchymal transition. Am J Respir Crit Care Med.

[24] Akdis CA, Bachert C, Cingi C, Dykewicz MS, Hellings PW, Naclerio RM, Schleimer RP, Ledford D (2013). Endotypes and phenotypes of chronic rhinosinusitis: a PRACTALL document of the European Academy of Allergy and Clinical Immunology and the American Academy of Allergy, Asthma & Immunology. J Allergy Clin Immunol.

[25] Gaffen SL (2009). Structure and signalling in the IL-17 receptor family. Nat Rev Immunol.

[26] Yu JJ, Gaffen SL (2008). Interleukin-17: a novel inflammatory cytokine that bridges innate and adaptive immunity. Front Biosci.

[27] Li G, Zhang Y, Qian Y, Zhang H, Guo S, Sunagawa M, Hisamitsu T, Liu Y (2013). Interleukin-17A promotes rheumatoid arthritis synoviocytes migration and invasion under hypoxia by increasing MMP2 and MMP9 expression through NF-kappaB/HIF-1alpha pathway. Molecular immunology.

[28] Straus DS (2013). TNFalpha and IL-17 cooperatively stimulate glucose metabolism and growth factor production in human colorectal cancer cells. Molecular cancer.

[29] Dang EV, Barbi J, Yang HY, Jinasena D, Yu H, Zheng Y, Bordman Z, Fu J, Kim Y, Yen HR, Luo W, Zeller K, Shimoda L (2011). Control of T(H)17/T(reg) balance by hypoxia-inducible factor 1. Cell.

[30] Shi LZ, Wang R, Huang G, Vogel P, Neale G, Green DR, Chi H (2011). HIF1alpha-dependent glycolytic pathway orchestrates a metabolic checkpoint for the differentiation of TH17 and Treg cells. J Exp Med.

[31] Shen Y, Tang XY, Yang YC, Ke X, Kou W, Pan CK, Hong SL (2011). Impaired balance of Th17/Treg in patients with nasal polyposis. Scand J Immunol.

[32] Li CW, Zhang KK, Li TY, Lin ZB, Li YY, Curotto de Lafaille MA, Shi L, Wang DY (2012). Expression profiles of regulatory and helper T-cell-associated genes in nasal polyposis. Allergy.

[33] Kim TH, Lee SH, Lee HM, Lee SH, Jung HH, Cho WS, Cinn YG, Choe H, Kim MP, Yoo IO, Hwang HY (2007). D2-40 immunohistochemical assessment of lymphangiogenesis in normal and edematous sinus mucosa and nasal polyp. Laryngoscope.

[34] Oakley GM, Curtin K, Orb Q, Schaefer C, Orlandi RR, Alt JA (2015). Familial risk of chronic rhinosinusitis with and without nasal polyposis: genetics or environment. International forum of allergy & rhinology.

[35] Meltzer EO, Hamilos DL, Hadley JA, Lanza DC, Marple BF, Nicklas RA, Bachert C, Baraniuk J, Baroody FM, Benninger MS, Brook I, Chowdhury BA, Druce HM (2004). Rhinosinusitis: establishing definitions for clinical research and patient care. J Allergy Clin Immunol.

[36] Cao PP, Li HB, Wang BF, Wang SB, You XJ, Cui YH, Wang DY, Desrosiers M, Liu Z (2009). Distinct immunopathologic characteristics of various types of chronic rhinosinusitis in adult Chinese. J Allergy Clin Immunol.

[37] Martin PM, Ananth S, Cresci G, Roon P, Smith S, Ganapathy V (2009). Expression and localization of GPR109A (PUMA-G/HM74A) mRNA and protein in mammalian retinal pigment epithelium. Molecular vision.

