# Supplementary material for: Hypoxia regulates IL-17A secretion from nasal polyp epithelial cells
Source: Oncotarget. 2017 Oct 31;8(60):102097–109. doi: 10.18632/oncotarget.22189 (PMC5731938; doi:10.18632/oncotarget.22189)
Supplement: Supplementary file 1 [file oncotarget-08-102097-s001.pdf]

Hypoxia regulates IL-17A secretion from nasal polyp epithelial cells

SUPPLEMENTARY MATERIALS

Immunohistochemistry

All monoclonal antibodies (anti-IL-17A, anti-HIF1α, anti-HIF2α, anti-IL-17A receptor, anti-IFN-γ, and anti-TSLP) were purchased from Abcam (Cambridge, MA, USA). An anti-IL-5 polyclonal antibody was purchased from Novus Biologicals. Primary antibodies were applied for 24 hours at 4°C, and slides were rinsed 3 times with phosphate-buffered saline (PBS) prior to incubation with the appropriate secondary antibodies for 2 hours. After further washing, the slides were stained with diaminobenzidine (DAB; Maixin, Fujian, Fuzhou, China) and counterstained with hematoxylin.

Nasal biopsies and primary nasal epithelial cell culture

Primary nasal polyps (NP) epithelial cells and IT(inferior turbinate) epithelial cells were freshly isolated from controls and patients with CRSwNP.

Biopsies of nasal epithelial cell sheets from the inferior turbinate of healthy volunteers were collected. After removing the mucus, the nostrils of volunteers were wide open with a sterilized rhinoscope and the inferior turbinate were scraped by interdental brushes (1.0 mm, diameter; GC, Suzhou, China). Brushes with biopsies were immediately transferred into PBS (Boster, Wuhan, China) containing 100mg/ml (Penicillin/Streptomycin) in sterilized tubes. The cells were centrifuged at 1000 rpm for 5 min and the supernatant was discarded. Nasal polyps tissue explants were washed and digested in 0.25% Trypsin (Gibco, Thermo Fisher scientific Inc,15050065) for 16~18 hour at 4°C. Cell suspensions were separated from particulate matter by centrifugation and resuspended in basal epithelial growth medium (BEGM) (Lonza, Walkersville, MD USA) with 10% FBS. Cells were plated for 2 h on 6cm^2 dishes(Thermo scientific, suzhou, China) to remove contaminating fibroblasts. Cells were then proliferated for seven to ten days on collagen-coated 3.5cm^{2}dishes (Thermo scientific, suzhou, China) in BEGM medium and starved off FBS. Once confluent, the cells were trypsinized and re-seeded evenly on human collagen type IV-coated (Sigma, USA, C5533) 3.5cm^2 dishes (Thermo scientific, suzhou, China). When cells were grown to 80% confluence, they were suspended in BEGM medium and further cultured for indicated time (0, 24 h, 48h) in HERA CELL 150i CO_2 incubator (Thermo scientific, USA) with appropriate oxygen concentrations for hypoxia (1% O2) or normoxia (21% O2).

Immunofluorescent staining and imaging

The secondary antibody was a goat anti-mouse antibody (Earthox, San Francisco, CA, USA). A 1 mg/mL DAPI solution (Solarbio Life Sciences, China) was applied to visualize nuclei. The plates were scanned, and images collected with an Operetta HTS imaging system (PerkinElmer) at 206x magnification with 12 fields of view (510*675 um)/well. Images were then analyzed using Columbus 2.2 software (PerkinElmer).

Quantitative RT-PCR

For single-stranded cDNA synthesis, 1μg (for cell lines) of total RNA was reverse transcribed using the RT-Systems supplied by Promega (Promega Corporation, Madison, USA). Quantitative real-time RT-PCR was carried out on a Master Cycler (Eppendorf).

The PCR of cytokines IL-5, IL-17A, IFN-γ, HIF1α was performed by using the SYBR Premix Ex Taq kit (TaKaRa Biotechnology, Dalian, China) with appropriate primers constructed from published sequences. Relative gene expression was calculated by using the comparative CT method. A 0 h hypoxia or normoxia exposure sample was used as a calibrator. β-actin was used as a housekeeping gene for normalization, and a no template sample was used as a negative control. The PCR primer sequences were as follows:

IL-5 forward	TCTACTCATCGAACTCTGCTGA
IL-5 reverse	CCCTTGCACAGTTTGACTCTC
IFN-y Forward	CCAACGCAAAGCAATACATGA
IFN-y Reverse	CCTTTTTCGCTTCCCTGTTTTA
HIF-1α forward	GAACGTCGAAAAGAAAAGTCTCG
HIF-1α reverse	CCTTATCAAGATGCGAACTCACA
IL-17A forward	AGATTACTACAACCGATCCACCT
IL-17A reverse	GGGGACAGAGTTCATGTGGTA

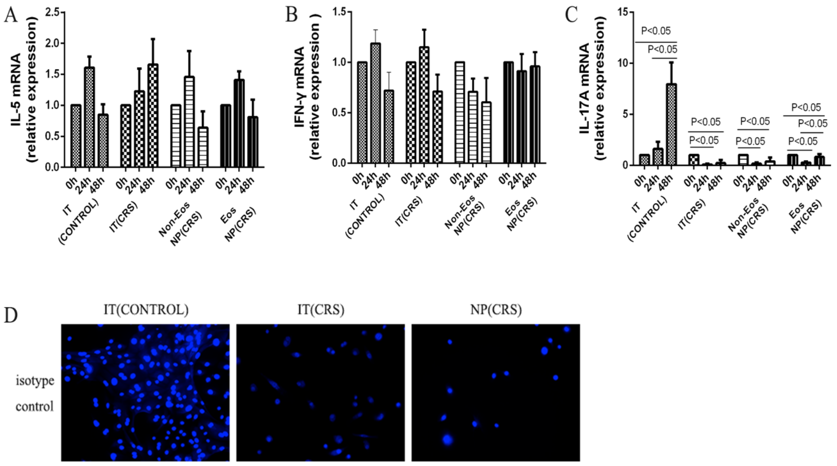

Supplementary Figure 1: The mRNA expression of IL-5, IFN-γ, IL-17A in control inferior turbinate (IT (CONTROL)), inferior turbinate of CRSwNP (IT (CRS)), eosinophilic (Eos) and noneosinophilic (Non-Eos) chronic rhinosinusitis with nasal polyps (CRSwNP) after exposure to hypoxia. **(A)** The mRNA expression of IL-5 in epithelial cells examined by quantitative RT-PCR. **(B)** The mRNA expression of IFN-y in epithelial cells examined by quantitative RT-PCR. **(C)** The mRNA expression of IL-17A in epithelial cells examined by quantitative RT-PCR. **(D)** High-content/high-throughput analysis isotype controls of IL-17A.
